# Reported Distribution Patterns of Five Kampo Formulations in the United States: Observations from a Single Manufacturer and Comparison with Japanese Prescribing Trends

**DOI:** 10.1177/27536130261488463

**Published:** 2026-09-09

**Authors:** Nathan L. Williams, Tetsuhiro Yoshino, Gregory Shumer, Kenji Watanabe, Tomonori Nakamura

**Affiliations:** 1 Benioff Children’s Hospital, 8785 University of California San Francisco, Oakland, CA, USA; 2 Graduate School of Pharmaceutical Sciences, 12913 Keio University, Tokyo, Japan; 3 Center for Kampo Medicine, 38084 Keio University School of Medicine, Tokyo, Japan; 4 Department of Family Medicine, 242909 University of Michigan, Ann Arbor, MI, USA; 5 68348 Faculty of Pharmaceutical Sciences, Yokohama University of Pharmacy, Yokohama, Kanagawa, Japan

**Keywords:** kampo, herbal medicine, traditional medicine, eastern medicine

## Abstract

**Background:**

Kampo medicine is a traditional Japanese herbal medical system that is integrated into contemporary healthcare in Japan but remains less widely characterized in the United States. Publicly available information regarding Kampo formulation utilization in the United States is limited.

**Methods:**

Manufacturer-reported distribution information was obtained through direct correspondence with Honso Pharmaceuticals, a company distributing Kampo formulations in the United States. The company provided a ranked list of its most commonly distributed Kampo formulations. The five highest-ranked formulations were reviewed descriptively and compared with published reports of Kampo prescribing patterns in Japan.

**Results:**

The five highest-ranked formulations reported by Honso Pharmaceuticals were Kamishoyosan, Shosaikoto, Hachimijiogan, Keishibukuryogan, and Juzentaihoto. These formulations differ from commonly prescribed Kampo formulations reported in Japanese health insurance and outpatient prescription datasets. Potential explanations may include differences in regulatory status, practitioner education, product availability, and healthcare-system structure between the United States and Japan.

**Conclusions:**

Manufacturer-reported distribution patterns suggest that Kampo formulation use in the United States may differ from prescribing trends reported in Japan. Because these observations are derived from a single manufacturer and lack patient-level utilization data, they should be interpreted cautiously. Additional research is needed to better characterize Kampo medicine utilization in the United States.

## Introduction

Kampo medicine is a traditional Japanese herbal medical system that is widely utilized there but poorly delineated elsewhere. It originated from medical knowledge introduced from China via the Korean peninsula and subsequently evolved within Japan.^1^ Today, Kampo medicine is integrated into the Japanese healthcare system where nearly 80% of physicians prescribe it alongside biomedical therapies. When prescribed by a physician, 148 Kampo formulations are reimbursed through Japan’s national health insurance system.^2^

In contrast, Kampo medicine remains unfamiliar to many healthcare professionals in the United States despite prevalent use of herbal supplements. According to the 2012 National Health Interview Survey, 17.9% of consumer respondents reported herbal medicine use in the past 12 months.^3^ A 2015 national survey found that as many as 35% of consumer respondents currently use at least one herbal preparation.^4^

Several studies have characterized Kampo prescribing patterns in Japan using national insurance claims and healthcare databases.^5,6^ However, publicly available information regarding Kampo formulation utilization or distribution patterns in the United States appears limited. To our knowledge, no national dataset describing Kampo formulation use in the United States has been reported.

The purpose of this brief report is to describe manufacturer-reported distribution patterns from a U.S.-facing distributor of Kampo formulations and compare these observations with published prescribing trends from Japan.

## Methods

### Data Source

Information regarding Kampo formulation distribution in the United States was obtained through an unpublished, personal communication with Honso Pharmaceuticals, a company that distributes Japanese Kampo formulations within the United States. In response to an academic inquiry from the authors, the company provided a ranked list of its most commonly distributed Kampo formulations in the United States.

Honso Pharmaceuticals was selected because it distributes Japanese Kampo formulations through a professional distribution network in the United States.^7^ Through its U.S. distribution partner, products are marketed primarily to licensed healthcare practitioners rather than through general consumer retail channels offering an exploratory proxy for professionally accessed Kampo products in the United States.

### Literature Review

A search of English-language literature was performed to identify commonly reported indications, safety considerations, and representative clinical studies associated with each formulation. Databases searched included PubMed and Google Scholar. Search terms included the English and romanized names of each Kampo formulation combined with terms such as “clinical trial,” “safety,” “adverse effects,” and “review.” This search was non-systematic and intended to provide contextual literature.

### Comparison With Japanese Prescribing Trends

Published studies describing Kampo prescribing patterns in Japan were reviewed and compared descriptively with the manufacturer-reported U.S. distribution rankings.

### Limitations of Data Source

No information regarding sales volume, market share, patient utilization, prescribing patterns, geographic distribution, methodology, or reporting period was provided by the manufacturer. Therefore, the findings should be interpreted as exploratory observations derived from a single manufacturer’s reported distribution rankings rather than a comprehensive assessment of Kampo utilization in the United States.

## Results

### Manufacturer-Reported Distribution Rankings

According to information provided by Honso Pharmaceuticals, the five highest-ranked Kampo formulations distributed within its United States network were Kamishoyosan, Shosaikoto, Hachimijiogan, Keishibukuryogan, and Juzentaihoto (Honso Pharmaceuticals, personal communication, January 15, 2024).

Published literature describing these formulations includes reports of applications related to menopausal symptoms, chronic liver disease, diabetic complications, vascular symptoms, and supportive oncology care. Representative indications, dosing approaches, and notable safety considerations identified from the literature are summarized in Table 1.

**Table 1.** Commonly Distributed Kampo Formulations From Honso Pharmaceuticals

Name	Representative clinical applications reported in literature	Reported dosage	Reported durations	Notable safety concerns
Kamishoyosan^*^	Management of hot flashes and symptoms associated with menopause^9^	7.5 g per day in three divided doses	A treatment duration of 8 weeks has been reported	Following ingestion geniposide, a compound within the gardenia fruit, rare case reports of idiopathic mesenteric phlebosclerosis (IMP) have been reported
Shosaikoto^*^	Management of chronic liver disease including hepatitis^10^	7.5 g per day in three divided doses	A treatment duration of at least 60 months (5 years) was described	Dose- and duration-dependent toxicity is possible, with interstitial lung disease and acute hepatitis reported. Contraindicated with concomitant interferon use, liver cirrhosis, and hepatic cancer^11^
Hachimijiogan	Management of diabetic sequelae including diabetic nephropathy^12^	7.5 g per day in three divided doses	A treatment duration of at least 6 months was described	Most common adverse effects include gastrointestinal symptoms and rash
Keishibukuryogan	Management of hot flashes and symptoms associated with menopause^9^	7.5 g per day in two divided doses (alternatively: 12.5 g per day in two divided doses)	A duration of 12 weeks was implemented	Low incidence of diarrhea as the most common adverse effect
Juzentaihoto^*^	Management of cancer and treatment-induced cachexia and immunocompromization^13^	7.5 g per day in three divided doses	A treatment duration of up to six years was evaluated	​

*Adverse event specific to licorice (glycyrrhiza root): excessive administration can cause pseudohyperaldosteronism and a constellation of associated symptoms including hypokalemia^8^.

## Discussion

### Distribution Patterns in the United States and Japan

The five highest-ranked formulations reported by Honso Pharmaceuticals differ from the Kampo formulations most commonly prescribed in Japan. Published analyses of Japanese health insurance and outpatient prescription data consistently identify formulations such as Kakkonto, Shoseiryuto, Maoto, Daikenchuto, Hochuekkito, and Shakuyakukanzoto among the most frequently prescribed Kampo medicines.^5,6^ Commercial sales data from Japan also suggest a different pattern of utilization. Tsumura & Co., which accounted for 84.6% of the Japanese prescription Kampo formulation market as of FY2025, reports Daikenchuto, Yokukansan, Goreisan, Hochuekkito, and Rikkunshito among its highest-ranked Kampo products.^14,15^ In contrast, none of these formulations appeared among the five highest-ranked products reported in the present U.S. dataset. Although direct comparison is limited by differences in data sources and healthcare systems, these observations suggest that Kampo distribution patterns in the United States may differ from those reported in Japan.

Several factors may contribute to the differences observed between the manufacturer-reported U.S. distribution rankings and published Japanese prescribing data. First, Kampo medicine occupies different roles within the healthcare systems of Japan and the United States. In Japan, Kampo formulations are prescribed by physicians, dispensed through pharmacies, and reimbursed through the national health insurance system.^16^ In contrast, Kampo products in the United States are generally regulated as dietary supplements and exist largely outside routine medical prescribing pathways.^17^

In Japan, Kampo formulations are manufactured under standardized quality controls, including Good Manufacturing Practice (GMP) and specifications outlined in the Japanese Pharmacopoeia.^18^ In the United States, products marketed as dietary supplements are regulated under a different framework and are not approved for disease treatment or prevention claims.^17^

Product availability may also differ between countries. For example, several commonly prescribed Kampo formulations in Japan contain ephedra (mao), an ingredient subject to significant regulatory restrictions in the United States.^19^

Finally, practitioner familiarity may vary substantially. Kampo medicine is incorporated into medical and pharmacy education in Japan,^16^ whereas formal instruction regarding Kampo remains uncommon in most American healthcare curricula.

Together, these clinical, regulatory, and educational differences represent plausible explanations for the observed differences, although their relative contributions cannot be determined from the available data. Future studies examining practitioner awareness, prescribing behaviors, and patient utilization patterns could help clarify the role of Kampo medicine within the United States healthcare landscape.

#### Limited Availability of U.S. Kampo Utilization Data

A notable observation arising from this study is the scarcity of publicly available information describing Kampo utilization in the United States. In Japan, multiple studies have examined Kampo prescribing patterns using national insurance claims and healthcare databases. To our knowledge, comparable datasets describing Kampo distribution or utilization in the United States have not been published.

This lack of publicly available information complicates efforts to characterize Kampo medicine use outside Japan and highlights an important gap in the literature. Consequently, manufacturer-reported distribution information may offer a preliminary perspective on Kampo activity within the United States despite its limitations.

## Limitations

This report has several important limitations. The findings were derived from information provided by a single manufacturer and therefore may not reflect broader Kampo utilization patterns within the United States. In addition, comparisons with published Japanese prescribing studies should be interpreted cautiously because those studies were based on national insurance claims and healthcare databases, whereas the present findings originated from manufacturer-reported distribution rankings.

The manufacturer provided inclusive data in response to an academic inquiry and did not disclose details regarding sales volume, market share, reporting methodology, ranking methodology, geographic distribution, practitioner characteristics, patient demographics, or indications for use. Furthermore, the data describe manufacturer-reported distribution rankings rather than actual patient utilization or prescribing patterns.

Accordingly, the present report should not be interpreted as identifying the most commonly used Kampo formulations in the United States. Rather, it provides an exploratory description of formulations reported by a single Kampo distributor and highlights the need for more comprehensive data regarding Kampo utilization in the United States.

## Conclusion

Publicly available information regarding Kampo formulation distribution in the United States remains limited. Manufacturer-reported rankings from a U.S.-facing Kampo distributor suggest distribution patterns that differ from prescribing trends reported in Japan. These differences may reflect variation in regulation, accessibility, practitioner familiarity, and broader healthcare-system structure. Additional research is needed to characterize Kampo utilization, users, and clinical applications within the United States.
